# The effectiveness of acupuncture in treating chronic non-specific low back pain: a systematic review of the literature

**DOI:** 10.1186/1749-799X-7-36

**Published:** 2012-10-30

**Authors:** Amanda J P Hutchinson, Simon Ball, Jeremy C H Andrews, Gareth G Jones

**Affiliations:** 1 Department of Physiotherapy, Trauma & Orthopaedics, Charing-Cross Hospital, Imperial College NHS Trust, Fulham Palace Road, London, W6 8RF, UK; 2 Department of Trauma & Orthopaedics, Chelsea and Westminster Hospital, 369 Fulham Road, London, SW10 9NH, UK; 3 Department of Anaesthetics & Critical Care, King's Mill Hospital, Mansfield Road, Sutton in Ashfield, Nottinghamshire, NG17 4JL, UK

## Abstract

**Background:**

Low back pain is a common musculoskeletal disorder defined as pain and soreness, muscle tension, or stiffness in the lumbosacral area of the spine which does not have a specific cause. Low back pain results in high health costs and incapacity to work causing an economic burden to society. The optimal management of non-specific low back pain appears to be undecided. Recently published guidelines support the use of acupuncture for treating non-specific low back pain and it has become a popular alternative treatment modality for patients with low back pain.

**Methods:**

A comprehensive systematic literature search was conducted through Medline using Ovid and Medical Subject Headings for randomized controlled trials published in the last 10 years. The outcomes scored were subjective pain scores and functional outcome scores.

**Results:**

Eighty two randomized studies were identified, of which 7 met our inclusion criteria. Three studies found a significant difference in pain scores when comparing acupuncture, or sham acupuncture, with conventional therapy or no care. Two studies demonstrated a significant difference between acupuncture treatment and no treatment or routine care at 8 weeks and 3 months. Three studies demonstrated no significant difference between acupuncture and minimal/sham acupuncture with no difference in pain relief or function over 6 to 12 months.

**Conclusions:**

This review provides some evidence to support acupuncture as more effective than no treatment, but no conclusions can be drawn about its effectiveness over other treatment modalities as the evidence is conflicting.

## Introduction

Low back pain is a common musculoskeletal disorder defined as pain and soreness, muscle tension, or stiffness in the lumbosacral area of the spine which does not have a specific cause [1]. Varying structures can contribute to these symptoms, including the joints, discs and connective tissues [1]. Disc degeneration and facet joint degeneration have been correlated with low back pain [2]. Approximately 90 percent of cases of low back pain are defined as non-specific [3]. Non-specific low back pain has no serious underlying pathology and no definable cause. It can also be classified into acute, sub acute and chronic. Most patients with acute low back pain recover quickly with 60-70% recovered by 6 weeks, but after 12 weeks recovery is slow and the development of chronicity occurs [4]. Chronic low back pain is defined as pain persisting more than 3 months [5].

Low back pain results in high health costs and incapacity to work causing an economic burden to society [6]. A national survey valign="top" carried out by the UK Department of Health in 1998 found that 40% of adults had suffered from back pain lasting more than a day in the previous 12 months [7]. This illustrated back pain to be the nation’s leading cause of disability with 1.1 million people disabled by it. In 2005, the British Trade Union Congress estimated 4.9 million working days lost due to low back pain [8]. One study estimated the total cost associated with the care and treatment of low back pain in the UK to be £1632 million with 37% related to physiotherapy and allied services, 31% to hospital care, 14% to primary valign="top" care, 7% to medication, 6% to community valign="top" care and 5% to radiology and imaging [9].

Evidence based guidelines for the management of low back pain have been published in a number of countries worldwide to try to improve patient outcomes [10]. There are many therapeutic treatments available but it is unclear from the literature which intervention is the most effective in treating nonspecific low back pain. Recently published NICE guidelines highlight the need for promotion of self-management and recommend a structured exercise programme, a course of manual therapy or a course of acupuncture of up to 10 sessions over 12 weeks [1]. Acupuncture has become a popular alternative treatment modality used by patients with low back pain and a course of acupuncture has been shown to be effective in relieving pain [11,12].

Acupuncture is based on the concepts of Chinese Medicine. The Chinese believe there are 12 main meridians in the body in which *Qi* energy must flow effectively through [13]. Fine gauge needles are inserted into certain points along these meridians activating the body’s natural healing. The needles are stimulated to achieve *De Q*i which is a feeling of warmth and heaviness [14]. Acupuncture is known to cause inhibition at the dorsal horn by activating the descending inhibitory pathways and stimulating release of opioids and serotonin [15]. Western acupuncture is an adaptation of Chinese acupuncture using some of the same classical points alongside extra needling points. Western acupuncture is practiced within the health service by doctors, physiotherapists and other healthcare practitioners [13].

This purpose of this study was to perform a review of the literature to assess the effectiveness of acupuncture in the treatment of adults with chronic non-specific low back pain.

## Methods

### Search strategy

A comprehensive literature search was conducted through Medline from 1950 to 2011 using OVID. Medical Subject Headings (MeSH) searched were “acupuncture therapy” or “acupuncture” or “acupuncture points”, and “treatment” or “therapeutics”, and “low back pain”. Boolean operators were used and the search was limited to randomized controlled trials published within the last 10 years in English.

### Inclusion criteria

Trials were included if they were randomized controlled trials within the last 10 years. The participants of interest were adults suffering from non-specific low back pain for 12 weeks or more with the study’s providing evaluation at three months or more. The treatments used were manual acupuncture which is the insertion of needles into acupuncture points along a meridian.

### Exclusion criteria

Trials were excluded if they were not randomized controlled trials, were duplicated studies, subject’s had low back pain of known origin (e.g. pregnancy, pain during labour or osteoporosis); compared different forms of acupuncture, used purely electro-acupuncture or auricular acupuncture or targeted a specific age group (e.g. the elderly) or analyzed cost effectiveness in isolation.

## Results

**Figure 1:**
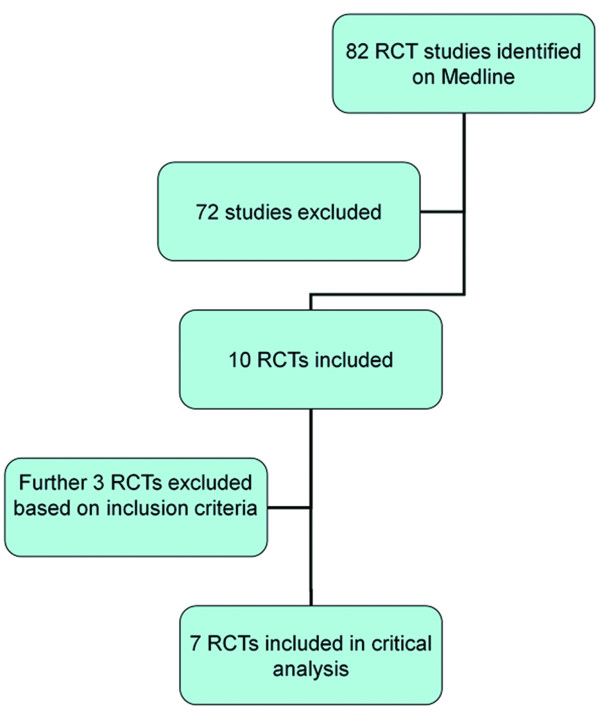
Number of studies identified and evaluated during the systematic review process.

The search identified 82 studies using the MeSH headings. The studies were screened initially and 72 studies were excluded as they looked at participants who were pregnant or elderly; the intervention was electro-acupuncture or they were non-randomized studies. Ten randomized controlled trials remained. On screening of these studies, 3 were excluded on their abstract, therefore 7 randomized controlled studies fulfilled our inclusion criteria (Figure 1).

There were a total of 13,874 participants with non-specific low back pain in these 7 studies evaluating manual acupuncture treatment with a control. The controls were minimal (sham) acupuncture, conventional treatment, placebo TENS and no treatment at all. The primary outcome measures used were Hannover Functional Ability Questionnaire (HFAQ), Von Korff Pain chronic pain scale, Visual Analogue Scale (VAS), Roland Morris Disability Questionnaire (RMDQ) and SF-36. Pain Disability Index, Oswestry Disability Index, SF-12, McGill present pain index, low back pain rating scale were examples of secondary outcome measures used. A summary of the details of studies are found in Table 1.

**Table 1:** Study valign="top" characteristics and critical appraisal of studies examining the effectiveness of acupuncture for low back pain

**Study**	**Intervention**	**Sample**	**Age**	**Duration of LBP**	**Outcome measures**	**Follow up**	**Results**
Haake et al. (2007) [17]	Acupuncture and Sham Acupuncture (x10-12, 30 minute sessions) and Guideline Based Conventional Therapy	1162	18- 86 years	> 6 months of non specific Low Back Pain	Von korff Chronic Pain Scale	6/52	Significant difference between acupuncture over conventional therapy. No significant difference between acupuncture and sham.
			Mean age 50 years			3/12	
					HFAQ	6/12	
					SF-12		
					1-6 scale of how good Treatment was		
					Medication use		
Witt et al. (2006) [16]	Manual acupuncture and no acupuncture control group and non randomized cohort.	11378	> 18 years	> 6 months of non specific Low Back Pain	HFAQ	3/12	Significant improvement in acupuncture group in back pain and function and cost effectiveness
			Mean age 52.9 years		SF-36	6/12	
					Low Back Pain Rating Scale		
	Maximum of 15 sessions						
					Cost effectiveness		
Brinkhaus et al. (2006) [18]	Manual acupuncture and Sham acupuncture using superficial acupoints (x12, 30 minute sessions over 8 weeks)	298	40-75 years	> 6 months of non specific Low Back Pain	VAS	8/52	Significant difference between acupuncture and no treatment. No difference between acupuncture and sham
					Pain Disability Index	26/52	
					SF-36	1 year	
					Emotional aspects of pain, depression, time with limited function/ pain/ analgesics taken		
	No Treatment.						
Thomas et al. (2006) [19]	Traditional manual acupuncture (10 sessions) Usual Care	241	18- 65 years	4-52 weeks of non specific Low Back Pain	SF-36	3/12	Significant difference at 24 months of small difference in the acupuncture group in the pain dimension of the SF-36.
					EuroQol	1 year	
					Oswestry Disability Index	2 years	
					McGill Pain Index		
					Analgesics		
Cherkin et al (2009) [20]	Individualised acupuncture (5-20 needles for 15-20 minutes), Standardized acupuncture (8 acupuncture points for 20 minutes), Simulated acupuncture (toothpick and needle guide), (All 10 sessions) Usual care	638	18-70 years	> 3 months non specific low back pain	Roland Morris Disability Questionnaire (RMDQ)	8/52	Significant difference between all acupuncture including individualized, standardized and simulated acupuncture and usual care in RMDQ at 8/52 and 26/52. No difference between acupuncture and sham.
						26/52	
					Bothersome Score	1 year	
					Physical and Mental health component of SF-36		
					Analgesics		
					Days spent in bed/ loss of work days		No difference at 1 year.
Kerr et al (2003) [21]	Standardized acupuncture (11 needles for 30 minutes, 10 sessions)	46	> 18 years	> 6 months	SF-36	6/52	Significant improvement in all outcomes for acupuncture. Significant improvement in SF-36, ROM and VAS for placebo TENS.
			Mean age 41.2 years		Lumber flexion ROM	6/12	
	Placebo TENS (4 electrodes, switched off, 30 minutes, 10 sessions)				Pain Rating Index (PRI) of the McGill Pain Questionnaire (MPQ)		
							No significant difference between the 2 groups.
					VAS		
Leibing et al (2002) [14]	Physiotherapy (26 sessions), Physiotherapy (26 sessions) and traditional and standardized acupuncture (20 sessions, needle insertion 10-30mm), Physiotherapy (26 sessions) and sham acupuncture (20 sessions, needle insertion 10-20mm)	131	18-65 years	> 6 months	Pain intensity (VAS)	12/52	Significant improvement in acupuncture group in all outcomes over control at 12/52. pain intensity (p<0.01), pain disability (p<0.01), Psychological distress (p<0.05). No significant difference in sham acupuncture and acupuncture.
					Pain Disability Index Hospital Anxiety and Depression Scale Lumbar spine flexion	1 year	

### The studies

Witt et al. recruited participants requesting acupuncture for the treatment of low back pain, who were insured by one of the participating social health insurance funds [16]. Eleven thousand, six hundred and thirty participants were randomly allocated into either the acupuncture group that received immediate acupuncture for 3 months or the control group who received delayed acupuncture after 3 months. Patients continued to receive routine care throughout the study. The outcome measures were HFAQ, SF-36 and low back pain rating scale measured at 3 and 6 months via questionnaires.

At 3 months mean HFAQ scores had increased by 12.1 points (15%) in the acupuncture group and by 2.7 points (3.5%) in the control group. The difference was 9.4 points (95% confidence interval) which was statistically significant (p < 0.01) showing acupuncture more effective than routine care. The SF-36 and Low Back pain rating scale were statistically significantly improved at 3 months in the acupuncture group compared to the control (p < 0.01). The non-randomized acupuncture group’s HFAQ increased by 14.6 points, 1.5 points more than the randomized acupuncture group (p < 0.01). After sub-analysis of the data acupuncture was seen to have a greater effect on patients with worse back function (p < 0.01) that were younger (p < 0.01). The changes at 6 months were slightly lower than at 3 months.

Haake et al. compared acupuncture treatment (needle insertion of 5 to 40 mm) with sham acupuncture (needle insertion of 1 to 3 mm) and a control of conventional therapy valign="top" consisting of physical therapy, exercise and drugs [17]. All participants had non-specific low back pain for more than 6 months and were aged over 18 years. The participants received ten, thirty minute sessions usually twice a week and an extra 5 sessions if they showed a 10-50% reduction in pain. One thousand one hundred and sixty two patients were randomized. The outcome measures were Von Korff chronic pain scale HFAQ and SF-12.

There were a variety of interventions given in the conventional therapy group including physiotherapy, massage, electrotherapy, injections and general exercise, of which some are more evidence based than others. There were statistically significant improvements in the acupuncture and sham acupuncture groups in their primary outcome measures (p < 0.01) over conventional therapy but no significant difference at 6 months between acupuncture and sham acupuncture (p = NS). The HFAQ scores and Von Korff pain scores improved in all three groups.

Brinkhaus et al. compared acupuncture treatment with minimal acupuncture (sham) and a control group of patients on a waiting list [18]. Participants were aged between 40 to 75 years with non-specific low back pain for 6 months or more. The acupuncture treatment was semi-standardized with the physicians using local and distal points to needle, with needles of unspecified length and stimulating the needles to achieve *De Qi* where possible. The minimal acupuncture group had needles inserted 20 to 40 mm, in predefined non acupuncture points, away from the lower back and these were not stimulated. The treatment in both groups consisted of twelve, thirty minute sessions over eight weeks. The outcome measures were Visual Analogue scale (VAS), Pain Disability Index and SF-36. Two hundred and ninety eight participants were randomized in a 2:1:1 ratio in favour of the acupuncture group. All completed questionnaires were sent directly to the study valign="top" centre. The VAS decreased by 28.7 mm (SD +/- 30.3 mm) in the acupuncture group at 8 weeks and by 23.6 mm (SD +/- 31.0 mm) in the minimal acupuncture group. The difference between acupuncture and minimal acupuncture was 5.1 mm (p = NS) and 21.77 mm between the acupuncture group and the waiting list group (p < 0.01). At 8 weeks there were significant differences between the acupuncture group and the waiting list group but not between the acupuncture and minimal acupuncture groups. The differences in outcome measures were reduced at 26 and 52 week follow ups. Comparison was difficult beyond 12 weeks as by then the control group had received acupuncture treatment.

Thomas et al. compared acupuncture treatment with usual care in patients with non specific low back pain of 4 to 52 weeks in duration, aged 18 to 65 years [19]. The acupuncture group received ten individualized sessions over 3 months. The acupuncturists determined the number of sessions and the contents of the sessions. There was no standardization of the acupuncture points used, the number of needles used, the duration of treatment, the depth of needle insertion or if the needles were manually stimulated. The usual care group was given National Health Service (NHS) treatment according to their general practioner’s assessment of their needs.

The primary outcome measure was the bodily pain dimension of the SF-36 at one year. A number of secondary outcome measures measuring pain again were also used, including Oswestry pain Disability Index, the McGill present pain index and the remaining dimensions of the SF-36. Follow up questionnaires were carried out by mail at 3, 12 and 24 months. Two hundred and forty one participants were randomized.

The acupuncture group received an average of 8.1 treatments. Patients in both groups received various other interventions. The results showed an intervention effect of 5.6 points (p = 0.06) in the SF-36 at 12 months and an estimated effect of 8.0 points (p < 0.01) at 24 months in the acupuncture group. No evidence of functional improvement was found and no data at 3 months is reported.

Cherkin et al. randomized 638 adults, with low back pain for more than 3 months, into individualized acupuncture, standardized acupuncture, simulated acupuncture or usual care [20]. Individualized acupuncture was prescribed by the diagnostician for each participant. Standardized acupuncture used set points prescribed by experts. Simulated acupuncture used a toothpick and guide tube. All participants underwent 10 treatment sessions. Outcome measures were assessed at 8, 26 and 52 weeks and included Roland Morris Disability Questionnaire, “Bothersome” Score and SF-36. There was a statistically significant improvement in function (RMDQ) in all groups at 8 weeks (p < 0.01) but was no longer significant at 52 weeks. The real and simulated acupuncture groups did not differ from each other (p = NS). At 8 weeks the proportion of patients improved in RMDQ scores was significantly greater in the real and simulated acupuncture groups compared with usual care (p < 0.01).

Kerr et al. randomly allocated 60 patients, with low back pain for more than 6 months, into 2 groups to receive either acupuncture or placebo TENS. Participants were treated weekly for 6 weeks with outcome measures including McGill Pain Questionnaire [21]. The VAS and SF-36 scores were collected pre and post treatment. Follow up was at 6 months. The acupuncture treatment was standardized using set points and 11 needles only for a duration of 30 minutes. Placebo TENS used 4 electrodes over the lumbar spine for 30 minutes. The machine was switched on but the circuit was broken so no current actually reached the patient. Both groups showed an improvement in their pre and post scores at 6 months. In the acupuncture group there was a significant difference in SF-36 (p < 0.01), MPQ (p < 0.01) and ROM (p < 0.01). There was a statistically significantly improvement in SF-36 for TENS placebo group (p < 0.01) and ROM (p < 0.05) and VAS (p < 0.05). There was no significant difference between the 2 groups for any outcome measure.

Leibing et al. randomly allocated 131 patients aged between 18 and 65 years with non-radiating low back pain of less than 6 months in duration [14]. All patients received 26 sessions of physiotherapy. The control group solely underwent physiotherapy, while the acupuncture group additionally received 20 sessions of traditional standardized acupuncture over 12 weeks. Fixed points were needled to a depth of 10-30 mm and stimulated to achieve *De Qi*. The sham acupuncture group 20 sessions of minimal acupuncture with needles inserted to a depth of 10-20 mm and not stimulated. Outcome measures were pain intensity, measured using a VAS, pain disability index, psychological distress, using the hospital anxiety and depression scale and lumbar spine flexion.

At 12 weeks acupuncture was superior to the control group in pain intensity (p < 0.01), pain disability (p < 0.01) and psychological distress (p < 0.05). There was no significant difference between acupuncture and sham acupuncture in pain disability or intensity although there was a difference in psychological distress (p < 0.05). At I year acupuncture was still superior to the control in pain disability (p < 0.05) but there were no differences between acupuncture and sham acupuncture. There were no differences in spine flexion throughout.

All 7 studies used outcome scores that assessed pain, disability and function. Witt et al demonstrated a statistically significant improvement in SF-36 (p < 0.01) and HFAQ (p < 0.01) scores at 3 months in the acupuncture group compared to routine care [16]. Cherkin et al similarly found a statistically significant increase in RMDQ scores in both the real and simulated acupuncture group (p < 0.01) over usual care at 8 weeks but there was no difference between real and simulated acupuncture (p = NS) [20]. Kerr et al showed a significant difference in the SF-36, McGill pain score and ROM in the acupuncture group when comparing pre and post scores at 6 months (p < 0.01) [21]. But this study revealed identical results for the placebo TENS patients with no difference in outcome scores between placebo TENS and acupuncture at 6 months. Thomas et al showed no functional improvement in acupuncture over usual care [19].

Brinkhaus et al revealed a significant reduction in VAS score for pain in the sham and acupuncture groups over patients on the waiting list at 8 weeks (p < 0.01) although there was no significant difference between the sham and acupuncture groups themselves [18]. The study by Haake et al supported these results stating a significant improvement in Von Korff pain scores in both the acupuncture and sham acupuncture over conventional therapy at 6 months (p < 0.01) [17]. As in the previous study there was no significant difference found between the sham and acupuncture groups (p = NS). The study by Leibing et al. [14] found similar results with a significant improvement in VAS scores at 12 weeks in the acupuncture group over the control (p < 0.01) which was the same at 1 year. Once again, there was no significant difference found between acupuncture and sham acupuncture.

## Discussion

This review provides some evidence to support acupuncture is better than no treatment, and some forms of conventional therapy, in providing some pain relief. The evidence from this review supports the theory that there is no significant difference between acupuncture and sham/ minimal acupuncture in providing pain relief and improvements in function.

Five studies [14,16-18,20] found a significant difference in their primary outcome measures- HFAQ, VAS, Roland Morris Disability Questionnaire, McGill Pain Index and Von Korff pain scores when comparing acupuncture, or sham acupuncture, with conventional therapy or no care. Two studies [16,18] demonstrated a significant difference between acupuncture treatment and no treatment or routine care at 8 weeks and 3 months. One study [16] found a significant improvement in pain, function and quality of life after acupuncture treatment compared to routine care at 3 months. Interestingly their study had a non-randomized arm consisting of participants refusing randomization, but requesting acupuncture. This group showed even greater improvements in these outcome measures which could support a degree of psychological response to treatment overestimating the effects of acupuncture. The improvement in pain in patients in the acupuncture group wasn’t significant enough for the patients to reduce the number of analgesics used during the trial as there was no significant difference between groups in analgesics being prescribed.

Two studies [17,19] showed conflicting evidence as to whether acupuncture was more effective than conventional treatment. Thomas et al. [19] showed minimal difference between acupuncture and usual care at 1 year, whereas Haake et al. [17] found acupuncture better than conventional therapy at 6 months. In both studies the control groups received a variety of interventions with some more evidence based than others making definitive conclusions about acupuncture’s effectiveness over such a broad range of therapies difficult to conclude. Due to high drop-out rates in the study by Thomas et al. [19], the study may have been underpowered to detect a significant difference.

Three studies [14,17,18] demonstrated no significant difference between acupuncture and minimal/sham acupuncture. There was no difference in pain relief or function over 6 to 12 months. Needle depth insertion varied between studies in both the acupuncture group and sham group. Haake et al. [17] inserted sham needles very superficially at 1 to 3 millimeters and the acupuncture group’s at 5 to 40 millimeters whereas Brinkhaus et al. [18] inserted the sham acupuncture needles into a greater depth of 20 to 40 millimeters mimicking the first studies treatment group. Leibing et al. inserted the acupuncture needles to a depth of 10-30 mm compared to 10-20 mm for the sham acupuncture. There appears to be no standardisation of the depth of needle insertion for true acupuncture compared to sham/ minimal acupuncture. The impact of this is difficult to assess as in practice different acupuncture points require different depth of needle insertion depending on their location. These 3 studies [14,17,18] stimulated the inserted needles in the acupuncture groups to achieve *De Qi*. All studies found no difference between the acupuncture treatment and the sham treatment questioning if stimulation of needles to achieve *De Qi* is necessary to be an effective treatment or indeed if needling specific points along a meridian is necessary, as needle insertion of minimal depth appears to have the same treatment effect.

There was limited standardization of needling techniques within the studies. Three studies [14,17,18] standardized the number of needles used. One study [18] used 6 to 10 needles in the sham acupuncture group and ten needles in the acupuncture group. The other study [17] used double the amount of needles at 14 to 20 per treatment in both groups. The details of the needling points used by the physicians were presented in only one study [18].

Despite there being no significant difference between acupuncture and sham acupuncture the evidence may advocate that needling soft tissue has a beneficial effect in relieving pain. Acupuncture could then be used as an adjunct to relieve pain, enabling patients to partake in a more rigorous treatment program for their lower back.

There was limited evidence from these studies to support the NICE guidelines recommendations of ten treatments over a twelve week period [1]. One study [17] carried out ten sessions in a five week period while two of the studies [16,18] offered weekly treatments providing up to 12 to 15 sessions although one study [16] identified the variation in the number of treatment sessions with some participants receiving 5 sessions compared to other participants in the same study receiving 15 sessions. These studies demonstrated a reduction in pain in the acupuncture groups compared to either no treatment or conventional therapy [16-18]. A fourth study [14] provided 20 sessions of acupuncture or sham acupuncture treatment over 12 weeks with sessions twice weekly initially. There was no evidence from these studies to support acupuncture providing long term pain relief benefit with all significant differences in pain being at 8 weeks to 3 months follow up. Recently published recommendations supported acupuncture as one treatment option for low back pain and suggested 10-12 acupuncture sessions over an 8 week period followed by a review of the patient’s pain, mood and general activity level prior to receiving further acupuncture sessions [2].

This review has limitations with only 7 studies reviewed and limited to publications in the last 10 years. There is a broad age variation of participants in these studies. Two studies reviewed participants 18 to 65 years of age [14,19] with the other study’s participants ranging up to 86 years of age. This disparity in age range may impact upon the results as those suffering low back pain at 55 years and over may be suffering from degenerative changes. Three studies [16-18] ensured the participants had non-specific low back pain for at least 6 months in duration. The fourth study’s participants were suffering low back pain from 4 to 52 weeks encompassing the sub-acute back pain sufferers with the chronic and those suffering additional leg pain [19]. There is great disparity in the methodologies and the treatment techniques used. The majority of these studies invited participants who wanted acupuncture treatment to join their studies potentially producing a selection bias. These participants may have brought positive expectations thereby influencing the results. The studies were limited to English studies when numerous research has been carried out in various countries such as China. The majority of outcome measures used in these studies were pain related rather a combination of pain scores and functional measures.

## Conclusion

This review provides some evidence to support acupuncture is more effective than no treatment but no conclusions can be drawn about its effectiveness over other treatment modalities as the evidence is conflicting. This review demonstrated sham acupuncture may be as effective as acupuncture which challenges the importance of needling along a meridian, the depth the needles need to be inserted and whether stimulation of the needles influences effectiveness of treatment. This review cannot provide guidance to the length of treatment sessions, the frequency of sessions, the number of needles needed or placement of needle insertion as there is great disparity in the acupuncture techniques used and no standardization of treatment.

In practice, acupuncture is rarely used in isolation but rather as an adjunct to other therapy modalities and this limited evidence supports this. There is a need for more research in this area to review acupuncture’s effectiveness as an adjunct to other therapy and compare the importance of needle placement, the depth of needle insertion, duration of treatment and the importance of needle stimulation to achieve *De Qi*. Trials using non-penetrating sham needles may be a more appropriate control.

## Competing interests

The authors declare that they have no competing interests.

## Authors’ contributions

Study valign="top" concept and design: AJPH, JB and GGJ. Acquisition of data: AJPH, SB and JCHA. Analysis and interpretation of data: AJPH, SB and JCHA. Drafting of the manuscript: AJPH, SB and JCHA. Critical revision of the manuscript for important intellectual content: AJPH and GGJ. Study supervision: AJPH. All authors read and approved the final manuscript.

## References

[B1] Early management of persistent non-specific low back pain: A NICE guideline http://guidance.nice.org.uk/CG88/NiceGuidance/pdf/English 20704057

[B2] Berman BM Langevin HM Witt CM Dubner R Acupuncture for chronic low back pain N Engl J Med 2010 363 454 461 10.1056/NEJMct0806114 20818865

[B3] Manek NJ MacGregor AJ Epidemiology of back disorders: prevalence, risk factors, and prognosis Curr Opin Rheumatol 2005 17 134 140 15711224 10.1097/01.bor.0000154215.08986.06

[B4] Andersson GB Epidemiological features of chronic low-back pain Lancet 1999 354 581 585 10.1016/S0140-6736(99)01312-4 10470716

[B5] Bogduk N Management of chronic low back pain Med J Aust 2004 180 79 83 14723591 10.5694/j.1326-5377.2004.tb05805.x

[B6] Maetzel A Li L The economic burden of low back pain: a review of studies published between 1996 and 2001 Best Pract Res Clin Rheumatol 2002 16 23 30 10.1053/berh.2001.0204 11987929

[B7] The prevalence of back pain in Great Britain in 1998 http://www.dh.gov.uk/en/Publicationsandstatistics/Publications/PublicationsStatistics/DH_4006687

[B8] 4.9 million lost work days is a pain in the back http://www.tuc.org.uk/workplace/tuc-10119-f0.cfm

[B9] Maniadakis N Gray A The economic burden of back pain in the UK Pain 2000 84 95 103 10.1016/S0304-3959(99)00187-6 10601677

[B10] van Tulder MW Tuut M Pennick V Bombardier C Assendelft WJ Quality of primary valign="top" care guidelines for acute low back pain Spine (Phila Pa 1976) 2004 29 E357 362 10.1097/01.brs.0000137056.64166.51 15534397

[B11] Carlsson CP Sjolund BH Acupuncture for chronic low back pain: a randomized placebo-controlled study with long-term follow-up Clin J Pain 2001 17 296 305 10.1097/00002508-200112000-00003 11783809

[B12] Sawazaki K Mukaino Y Kinoshita F Honda T Mohara O Sakuraba H Togo T Yokoyama K Acupuncture can reduce perceived pain, mood disturbances and medical expenses related to low back pain among factory employees Ind Health 2008 46 336 340 10.2486/indhealth.46.336 18716381

[B13] White A Western medical acupuncture: a definition Acupunct Med 2009 27 33 35 19369193 10.1136/aim.2008.000372

[B14] Leibing E Leonhardt U Koster G Goerlitz A Rosenfeldt JA Hilgers R Ramadori G Acupuncture treatment of chronic low-back pain – a randomized, blinded, placebo-controlled trial with 9-month follow-up Pain 2002 96 189 196 10.1016/S0304-3959(01)00444-4 11932074

[B15] Vickers A Zollman C ABC of complementary medicine acupuncture BMJ 1999 319 973 976 10.1136/bmj.319.7215.973 10514163 PMC1116804

[B16] Witt CM Jena S Selim D Brinkhaus B Reinhold T Wruck K Liecker B Linde K Wegscheider K Willich SN Pragmatic randomized trial evaluating the clinical and economic effectiveness of acupuncture for chronic low back pain Am J Epidemiol 2006 164 487 496 10.1093/aje/kwj224 16798792

[B17] Haake M Muller HH Schade-Brittinger C Basler HD Schafer H Maier C Endres HG Trampisch HJ Molsberger A German Acupuncture Trials (GERAC) for chronic low back pain: randomized, multicenter, blinded, parallel-group trial with 3 groups Arch Intern Med 2007 167 1892 1898 10.1001/Archinte.167.17.1892 17893311

[B18] Brinkhaus B Witt CM Jena S Linde K Streng A Wagenpfeil S Irnich D Walther HU Melchart D Willich SN Acupuncture in patients with chronic low back pain: a randomized controlled trial Arch Intern Med 2006 166 450 457 16505266 10.1001/archinte.166.4.450

[B19] Thomas KJ MacPherson H Thorpe L Brazier J Fitter M Campbell MJ Roman M Walters SJ Nicholl J Randomised controlled trial of a short course of traditional acupuncture compared with usual care for persistent non-specific low back pain BMJ 2006 333 623 10.1136/bmj.38878.907361.7C 16980316 PMC1570824

[B20] Cherkin DC Sherman KJ Avins AL Erro JH Ichikawa L Barlow WE Delaney K Hawkes R Hamilton L Pressman A A randomized trial comparing acupuncture, simulated acupuncture, and usual care for chronic low back pain Arch Intern Med 2009 169 858 866 10.1001/archinternmed.2009.65 19433697 PMC2832641

[B21] Kerr DP Walsh DM Baxter D Acupuncture in the management of chronic low back pain: a blinded randomized controlled trial Clin J Pain 2003 19 364 370 10.1097/00002508-200311000-00004 14600536

